# CDC light traps underestimate the protective efficacy of an indoor spatial repellent against bites from wild *Anopheles arabiensis* mosquitoes in Tanzania

**DOI:** 10.1186/s12936-023-04568-5

**Published:** 2023-04-29

**Authors:** Johnson Kyeba Swai, Ummi Abdul Kibondo, Watson Samuel Ntabaliba, Hassan Ahamad Ngoyani, Noely Otto Makungwa, Antony Pius Mseka, Madeleine Rose Chura, Thomas Michael Mascari, Sarah Jane Moore

**Affiliations:** 1 Vector Control Product Testing unit, Environmental Health and Ecological Science Department, Ifakara Health Institute, Bagamoyo, Tanzania; 2 Swiss Tropical and Public Health Institute, Allschwil, Switzerland; 3 University of Basel, Basel, Switzerland; 4 S. C. Johnson & Son, Inc., Racine, WI USA; 5 The Nelson Mandela, African Institution of Science and Technology, School of Life Sciences and Bio Engineering, Tengeru, Arusha, United Republic of Tanzania

**Keywords:** Spatial repellent, Volatile pyrethroid, *Anopheles*, Mosquito sampling, Protective efficacy, Vector control

## Abstract

**Background:**

Methods for evaluating efficacy of core malaria interventions in experimental and operational settings are well established but gaps exist for spatial repellents (SR). The objective of this study was to compare three different techniques: (1) collection of blood-fed mosquitoes (feeding), (2) human landing catch (HLC), and (3) CDC light trap (CDC-LT) collections for measuring the indoor protective efficacy (PE) of the volatile pyrethroid SR product Mosquito Shield^™^

**Methods:**

The PE of Mosquito Shield^™^ against a wild population of pyrethroid-resistant *Anopheles arabiensis* mosquitoes was determined via feeding, HLC, or CDC-LT using four simultaneous 3 by 3 Latin squares (LS) run using 12 experimental huts in Tanzania. On any given night each technique was assigned to two huts with control and two huts with treatment. The LS were run twice over 18 nights to give a sample size of 72 replicates for each technique. Data were analysed by negative binomial regression.

**Results:**

The PE of Mosquito Shield^™^ measured as feeding inhibition was 84% (95% confidence interval (CI) 58–94% [Incidence Rate Ratio (IRR) 0.16 (0.06–0.42), p < 0.001]; landing inhibition 77% [64–86%, (IRR 0.23 (0.14–0.36) p < 0.001]; and reduction in numbers collected by CDC-LT 30% (0–56%) [IRR 0.70 (0.44–1.0) p = 0.160]. Analysis of the agreement of the PE measured by each technique relative to HLC indicated no statistical difference in PE measured by feeding inhibition and landing inhibition [IRR 0.73 (0.25–2.12) p = 0.568], but a significant difference in PE measured by CDC-LT and landing inhibition [IRR 3.13 (1.57–6.26) p = 0.001].

**Conclusion:**

HLC gave a similar estimate of PE of Mosquito Shield^™^ against *An. arabiensis* mosquitoes when compared to measuring blood-feeding directly, while CDC-LT underestimated PE relative to the other techniques. The results of this study indicate that CDC-LT could not effectively estimate PE of the indoor spatial repellent in this setting. It is critical to first evaluate the use of CDC-LT (and other tools) in local settings prior to their use in entomological studies when evaluating the impact of indoor SR to ensure that they reflect the true PE of the intervention.

## Background

Incremental reductions to the burden of malaria will require new vector control tools beyond the core tools: insecticide treated nets (ITN) and indoor residual spray (IRS) [1]. The efficacy of new tools must be demonstrated using techniques that are appropriate both in terms of relatedness to entomological endpoints relevant to disease transmission as well as feasibility of implementation in experimental and operational contexts. Protocols and methods for evaluating ITNs and IRS have been well established to measure efficacy in both controlled experimental settings [2, 3] and operationally [4, 5], but there are gaps in guidance for other vector control interventions such as spatial repellents (SR) [6, 7].

There are numerous existing spatial repellents (SR) products including coils, liquid vaporizers, heated mats, and ambient emanators, that reach millions of end users globally through commercial channels. These can be used both indoors and outdoors to prevent mosquito bites [8, 9]. Many national regulatory authorities have detailed laboratory methods and guidelines in place for evaluating the efficacy of SR products, which manufacturers use to generate data for dossier submissions in support of product registrations (e.g., United States Environmental Protection Agency (US EPA) [10], Biocidal Products Regulation (BPR) [11], Malaysia Standard). However, SR currently do not have a policy recommendation from the World Health Organization (WHO) for use against malaria, although there is growing evidence of the public health benefit of SR products [12, 13]. Therefore, guidance on methods for measuring efficacy in experimental and operational settings is needed.

The key entomological endpoint impacted by SR is blood-feeding [14–17] though many other impacts against mosquitoes have been described experimentally, such as landing inhibition, repellency, excito-repellency, knock down, disarming, mortality and effects on fertility and fecundity [16, 18–21]. The most direct means of showing the impact of SR on blood-feeding is through the collection of blood-fed mosquitoes, which may be done experimentally in huts designed to allow mosquitoes to enter (but not exit) and feed on the human study participants sleeping inside as is commonly done during the evaluation of ITNs [3] and IRS [2]. Protective efficacy of SR applied indoors can be calculated as the proportional reduction in blood-feeding rates or in the number of blood-fed mosquitoes versus a negative control [6]. While assessment of reductions in blood-feeding can be measured in end user homes by indoor resting collections [13], this method is difficult to implement cost effectively due to the low numbers of blood fed mosquitoes recovered.

Human landing catch (HLC) is commonly used to calculate human biting rates (HBR) and is identified as a method to evaluate protective efficacy of a SR through the calculation of proportional reductions of lands in a treatment versus a negative control [6, 7]. Human landing catch is more broadly usable than methods used to measure blood-feeding rates directly, and can be conducted successfully in controlled laboratory settings, outdoor experimental huts, or end user homes [22]. Mosquito lands are conceptually linked to blood-feeding, and previous research shows there is a relationship between the blood-feeding rates and lands [23]. However, SR interfere with mosquito host-seeking capabilities by affecting their olfactory receptors [20], and it is possible that not all mosquitoes that land are able to feed [16] potentially underestimating the protective efficacy (PE) that would be measured by blood-feeding inhibition.

The CDC light trap (CDC-LT) has been used as a tool to approximate HBR, and a large body of evidence exists on comparing collections of malaria vectors through HLC and CDC-LT [24–27]. The CDC-LT may provide some logistical advantages over HLC in operational settings (ease of use) with no increased risk of exposure of study participants to mosquito bites [24, 25] although it does not generally compare directly to HLC [25, 26, 28]. However, it is currently unknown if the CDC-LT is an appropriate tool to measure reductions in the HBR by SR applied indoors i.e., whether the proportional reduction in mosquitoes captured by light traps is a suitable proxy for proportional reductions in blood-feeding or lands.

The objective of this study was to compare PE estimates of Mosquito Shield^™^ against a wild population of *Anopheles arabiensis* from direct measurement of blood-feeding, HLC, or CDC-LT.

## Methods

### Study location

The study was conducted from November to December 2021 at the Ifakara Health Institute (IHI) Field Station located in Lupiro village (8.385°S and 36.670°E) in Ulanga District, south-eastern Tanzania. The village lies 270 m above sea level on the Kilombero River valley, south of Ifakara town. Lupiro borders many small contiguous and perennially swampy rice fields to the northern and eastern sides. The annual rainfall is 1200–1800 mm with temperatures ranging between 20 and 33 °C. The main malaria vectors include *An. arabiensis* and *Anopheles funestus *sensu stricto both of which are resistant to pyrethroids [29, 30]. *Anopheles funestus* mediates most of the transmission [31–33]. ITNs are the main vector control tool in the region, and are mass distributed by the Government [34].

### Experimental huts

This study was conducted in Ifakara experimental huts [35], with some modifications. The modifications included a division of the huts into two 3.5 × 3.25 m rooms, each with its own entrance and two exit traps. These rooms, hereafter referred to as individual huts, were much closer in size to the other experimental hut types (west African and east African) [36]. A total of 12 huts were used to run the experiment, six with treatment and six with control.

### Intervention

Mosquito Shield™ is a folded 21.6 cm × 26.7 cm sheet of plastic film dosed with 110 mg of transfluthrin, with a label claim of 30 days duration (SC Johnson & Son, Racine, WI, USA). A total of four Mosquito Shield^™^ products were placed in each hut according to manufacturer’s instructions (at a height of 1.5 m from the ground and at centre length of each wall). The Mosquito Shield^™^ products were installed at 16:00 h on the first day of the study and were not removed until the last day (18 days).

### Study design

**Fig. 1:**
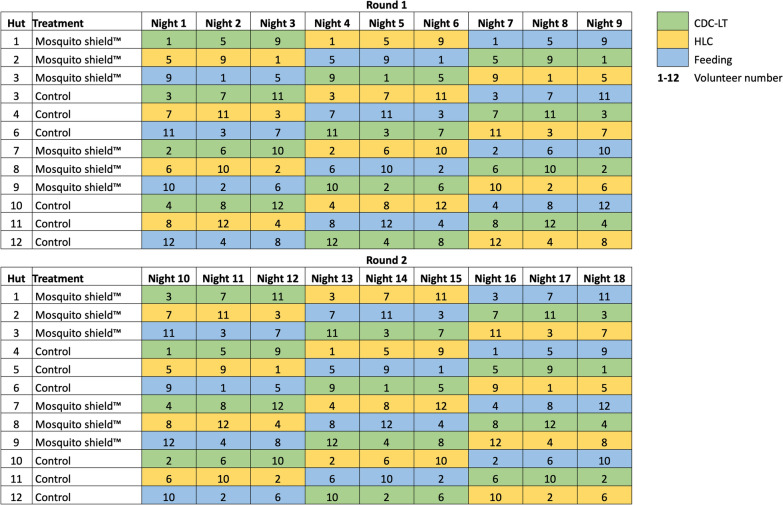
Latin square rotation of the collection methods (Feeding, HLC and CDC-LT) and study volunteers in the set of huts with the Mosquito Shield^™^ and control (no treatment)

The performance of three different techniques (feeding, HLC, CDC-LT) in estimating the efficacy of Mosquito Shield^™^ was evaluated in 12 experimental huts: 6 assigned to control and six assigned to treatment. Four simultaneous 3 by 3 Latin squares (LS), two LS in the control arm and two in the treatment arm, were conducted twice over a total of 18 nights (Fig. 1). Twelve male volunteers participated in the study due to the risk of malaria in pregnancy and cultural norms of Tanzania. Three volunteers were fixed to each LS and rotated each night. Volunteers assigned to the control arm for the first LS were assigned to the treatment arm in the second LS and vice versa (Fig. 1). The techniques were randomly allocated to huts using a random number generator, and after every third night they rotated to a different set of huts. In this way, on any given night each technique was assigned to two huts with control and two huts with treatment, and each volunteer tested each treatment with each technique 9 times.

Both huts within a single original Ifakara experimental hut received the same treatment throughout the duration of the study (i.e., either four Mosquito Shield™ products or negative control) to ensure no treatment interactions occurred between contiguous huts. In addition, huts were approximately 20 m apart from each other to ensure independence of observations. Every morning, the hut doors and windows were closed and at 16:00 h the windows were opened to allow ventilation in the huts. This was done in the CDC-LT and HLC huts only since there were no window exit traps, while window exit traps were used for the feeding technique to recapture fed mosquitoes. Mosquitoes collected were identified to species level using morphological keys [37].

### Mosquito collection techniques

**Fig. 2:**
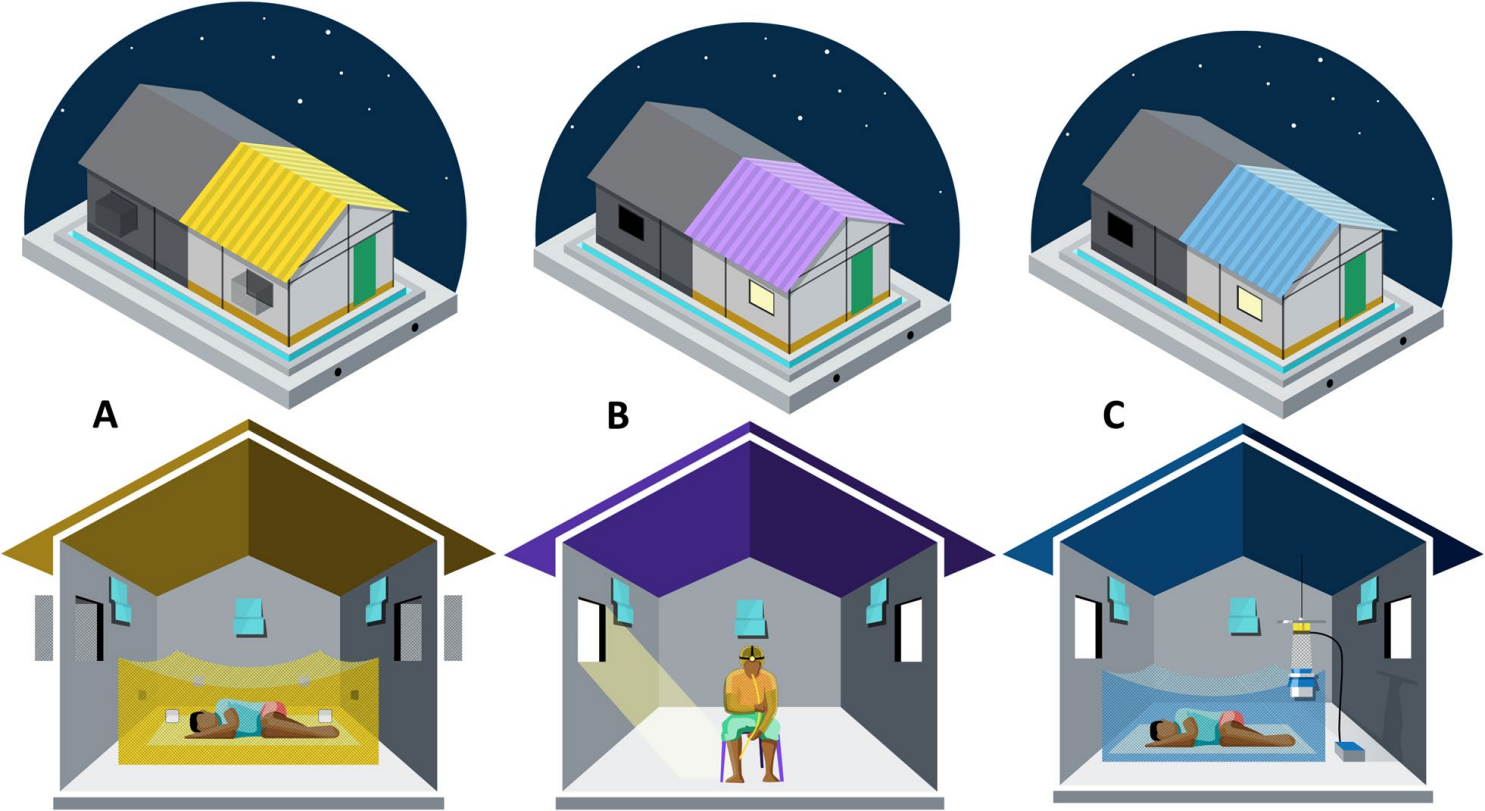
Set up of huts used for “Feeding” (**A**), Human Landing Catch “Landing” (**B**) and CDC-LT (**C**) experiments including the placement of the Mosquito Shield^™^

For the feeding technique the volunteers slept under an untreated bed net (SafiNet^™^, A to Z Textile Mills, Ltd., Arusha, Tanzania) inside the huts from 18:00 to 07:00 h each night (Fig. 2). Nets were deliberately holed with eight 4 × 4 cm holes: two on the roof, one on each short side and two on each long side of the nets to simulate a damaged bed net. In the morning, mosquitoes were collected from inside the bed net, and window exit traps using mouth aspirators and from the floor and walls of the hut using prokopack aspirators. These mosquitoes were then taken to the field laboratory that is located near the experimental hut site, placed in a freezer to be killed, then sorted and scored by species, physiological status, collection location and hut.

For the HLC technique, collections were performed inside the huts from 18:00 to 07:00 h. A volunteer sat on a chair placed in the centre of each hut, wearing shorts, closed shoes and a net jacket to prevent mosquitoes biting on the feet or above the knees. The volunteers caught mosquitoes landing on their exposed lower legs for 50 min periods per hour using mouth aspirators and torch lights. At the top of each hour volunteers took a break to maintain alertness. Live mosquitoes collected were placed in small paper cups and the following morning were taken to a freezer in the field laboratory that is located near the experimental hut site, to be killed before being sorted and scored by species, collection hour, and hut.

For the CDC-LT technique, the volunteers slept under intact untreated bed nets inside the huts from 18:00 to 07:00 h each night. A CDC-LT trap was hung 1 m above the ground adjacent to the foot end of the sleeping space [38]. In the morning, mosquitoes in the CDC-LT were collected as well as from inside the hut using prokopack aspirators. The mosquitoes were then taken to a freezer the field laboratory that is located near the experimental hut site to be killed before being sorted and scored by species, physiological status, collection location and hut. Only mosquitoes collected by CDC-LT were used in the analysis.

### Data analysis

Analysis was performed using STATA 16 software (StataCorp LLC, USA). Descriptive statistics were presented as Williams means [39] of nightly collections with 95% confidence intervals (95% CI). Williams mean was used because mosquito count data was highly skewed [40]. It was calculated by [(geometric mean of (x + δ))- δ, when δ =1].

Protective efficacy (PE) was the primary outcome measure for each technique. PE was defined as the reduction in the number in the treatment relative to the control. For feeding, PE was defined as feeding inhibition i.e., the reduction in the number of fed mosquitoes recaptured in the experimental hut; for HLC PE was defined as landing inhibition i.e., the reduction in the number of landed mosquitoes; for CDC-LT PE was defined as reduction in numbers collected by light trap. The effect of the treatment on the nightly collections for each technique was examined using generalized linear regression with a negative binomial distribution with a log link. The data was modelled with treatment, and night as fixed factors for each technique. The PE for each experiment was calculated by (1-IRR) *100, where IRR is the incidence risk ratio in the Mosquito Shield™ group compared to the negative control. Each technique was analysed separately to measure PE. Additionally, the agreement between the experimental methods in estimating PE was explored using the same regression model with an interaction between treatment and technique.

## Results

A total of 3755 *An. arabiensis* were collected and used for analysis in this study: 50 (1.3%) from feeding technique, 2151 (57.3%) from HLC, and 1554 (41.4%) from CDC-LT.

In the feeding method, fewer mosquitoes were collected in the treatment than the control arm. This was significantly different (IRR 0.16 (0.06–0.42) P < 0.0001), Table 1. The PE was estimated to be 84% (58–94%). The Williams mean number of blood-fed *An. arabiensis* mosquitoes collected in Mosquito Shield™ and negative control huts had overlapping 95% confidence intervals. This is likely due to high variability in the numbers of mosquitoes collected as the number of blood fed mosquitoes was low.

**Table 1:** Estimates of protective efficacy of Mosquito Shield^™^ against wild *Anopheles arabiensis* using different techniques

Technique	Outcome measures	Treatment	Total captured^a^	William’s mean^b^ (95% CI)	% PE (95% CI)	IRR (95%CI)	P-value
Feeding	Feeding inhibition	Negative control	43	11.8 (6.1–17.6)	_	1	< 0.0001
		Mosquito shield^™^	7	3.1 (0–6.4)	84% (58–94)	0.16 (0.06–0.42)	
HLC	Landing inhibition	Negative control	1724	38.0 (30.6–47.2)	_	1	< 0.0001
		Mosquito shield^™^	427	9.6 (7.4–12.3)	77% (64–86)	0.23 (0.14–0.36)	
CDC-LT	Reduction in numbers collected	Negative control	896	19.1 (14.1–25.7)	_	1	0.160
		Mosquito shield^™^	658	14.1 (10.5–18.9)	30% (0–56)	0.70 (0.44–1.0)	

^a^Total captured refers to total number of blood-fed *An. arabiensis* collected for Feeding technique; total number of landed *An. arabiensis* for HLC technique; total number of *An. arabiensis* collected in CDC-LT for CDC-LT

^b^William’s mean  = [(geometric mean of (x + δ))-δ, when δ = 1]. PE (protective efficacy) = 1-IRR estimates

Similarly, for the HLC technique fewer mosquitoes were collected in the treatment than the control arm and this was significantly different (IRR 0.23 (0.14–0.36) P < 0.0001), Table 1. The PE was estimated to be 77% (64–86%). The Williams mean number of *An. arabiensis* mosquitoes collected in Mosquito Shield^™^ and negative control huts had non-overlapping 95% confidence intervals as HLC collected a higher number of landed mosquitoes overall, and consequently estimates were more precise.

For the CDC-LT technique, although the treatment arm had fewer mosquitoes than the control arm, the difference was not significantly different [IRR 0.70 (0.44–1.0) P = 0.160], Table 1. The PE was estimated to be 30% (0–56%) with wide confidence intervals. The Williams mean number of blood-fed *An. arabiensis* mosquitoes collected in Mosquito Shield^™^ and negative control huts had widely overlapping 95% confidence intervals as the estimate of mosquito density in the treatment and control arms was similar.

Analysis of the interaction between treatment and technique indicated that there was no significant difference in PE calculated using feeding inhibition or using landing inhibition [IRR 0.73 (0.25–2.12), P = 0.568] (Table 2). Protective efficacy calculated by CDC-LT catches was significantly different from that estimated by HLC [IRR 3.13 (1.57–6.26), P = 0.001]. Relative to HLC, the CDC-LT collected more mosquitoes in the treatment arm and fewer in the control arm whereas FI and HLC both showed a consistent direction of effect with fewer mosquitoes in both the control and treatment arms for FI.

**Table 2:** Comparison of the different data collection methods in estimating protective efficacy against wild *Anopheles arabiensis*

	Method	IRR (95% CI)	P-value
Overall difference between collection methods	HLC	1	
	Feeding	0.73 (0.25–2.12)	0.568
	CDC-LT	3.13 (1.57–6.26)	0.001
Within treatment	HLC	1	
	Feeding	0.01 (0.01–0.04)	< 0.0001
	CDC-LT	1.43 (0.87–2.33)	0.158
Within control	HLC	1	
	Feeding	0.02 (0.01–0.04)	< 0.0001
	CDC-LT	0.48 (0.29–0.80)	0.005

The reference is set as HLC for each comparison

*HLC* Human landing catch, *CDC-LT* Centers for disease Control and prevention miniature light traps, *IRR* Incidence rate ratio from negative binomial regression

## Discussion

The objective of this study was to compare three different techniques for measuring PE of Mosquito Shield^™^ against *An. arabiensis* mosquitoes: direct measurement of blood-feeding, HLC, or CDC-LT. We found that PE estimated from feeding and HLC were similar in magnitude, and were not statistically different, while PE measured using CDC-LT was around half of that measured by HLC or feeding and differed in measurement to a statistically significantly degree. The conclusion from this study is that CDC-LT could not effectively estimate the PE of the indoor SR in this setting.

This study presents an evidence-based position on the inability of CDC-LT to accurately measure the entomological impact of SR against malaria vectors in Tanzania. Previous studies in the region compared efficacies of HLC and CDC-LT for mosquito surveillance but not for evaluating efficacy of an intervention [24, 27, 41]. The study was conducted in one geographical location with results against one malaria vector species, and we acknowledge that it is possible that CDC-LT could be appropriate for other settings. However, it is clear from this study that it cannot be expected without evidence that CDC-LT, while a ubiquitous and convenient tool, is appropriate for all entomological research questions, including evaluation of spatial repellents. There is a large amount of data that demonstrates that CDC-LT is a valuable tool for measuring the indoor density of host seeking mosquitoes [25, 26] but not necessarily human exposure to mosquitoes [28].

A repellent was defined by Browne as causing prevention of mosquitoes reaching a source to which they would otherwise be attracted [42] which can occur by taxis, kinesis, inhibition of attraction [43] or sublethal incapacitation [18]. It is possible that these modes of action are not well captured by the CDC-LT that estimates indoor densities of mosquitoes. It is also possible that the pyrethroid used in the spatial repellent affected the catch in the CDC LT. This was observed in other studies from Tanzania where CDC-LT placed next to ITNs captured more mosquitoes than those next to untreated ITNs [44]. This was hypothesised to be due to excito-repellency driving mosquitoes towards the light used in the CDC LT. A test of metofluthrin SR in Cambodia using CDC-LT in the absence of a human sleeper, placed under houses showed a reduction *in Anopheles* but not *Culex* catches, and it was again hypothesised that light used in the CDC-LT may have played a role in the inconsistency of the results observed [45].

Despite the suitability of direct measurement of blood-feeding and HLC in measuring PE of indoor SR, there are constraints on their use in some contexts. Direct measurement of blood-feeding may not be feasible for in-home tests and in operational settings due to ethical considerations around increased exposure to disease-carrying mosquitoes and the challenge of consistently capturing all mosquitoes that blood-feed indoors. Use of HLC can also have additional safety considerations due to possible increased risk of exposure to vectors [46, 47] although medically supervised HLC mitigates much of this risk [48]. Nonetheless. it can be labour intensive and taxing on volunteers when done at large scale [26, 49], and can be challenging to standardize due to differences in human attractiveness to mosquitoes, skilfulness of collectors, and alertness throughout the collection period [25, 26]. Evaluations of the Mosquito Electrocuting Trap (MET) and Biogents Sentinel Trap (BGS) for measurement of the PE of SR using *Aedes aegypti* have also shown some promise in semi-field experiments if observations are independent because it was observed that mosquitoes divert from traps to nearby humans [50].

Use of CDC-LT has the advantage of having lower risk of exposure to disease vectors for volunteers relative to direct measurement of blood-feeding or HLC but was shown in this study to not be a viable alternative for estimation of the PE of SR. Several other lower-exposure sampling techniques including Suna^®^ trap [51], mosquito-electrocuting trap (MET) [52–54], miniaturized double-net trap (DN-Mini) [55] and human baited double net trap (HDN) [56] have been developed and tested for surveillance and control of host-seeking mosquitoes [25, 51–54, 57]. Further research is warranted to evaluate these traps or find alternatives to HLC that are appropriate for measuring PE of indoor SR.

## Limitations

One possible limitation to this study is that we assumed that all blood-fed mosquitoes had fed on study volunteers even though blood-meal identification was not conducted. While it is our position that this was very unlikely as there were no animal sheds near the study area and many blood fed mosquitoes were collected from inside of the damaged untreated bednets, if bloodmeals were taken from alternative hosts, comparisons between techniques could have been impacted.

Another limitation was that the study was done in experimental huts, which could mean the results are not identical to what would have been observed in an in-home test in the same area. However, it is unlikely that CDC-LT would give an estimate of PE closer to that of HLC in homes than in a controlled setting like an experimental hut. We decided to run this study in experimental huts to reduce confounding factors including differential number and type of mosquito entry points, home size and construction materials, and environmental conditions within homes that may influence emanation rates of the SR. The use of huts also ensures independence of observations as only one individual is present in each hut.

### Next steps

As part of an ongoing large-scale clinical trial evaluating Mosquito Shield^™^ in western Kenya, monthly CDC-LT collections and quarterly collection via HLC are being conducted over a 2 year period. This may allow further comparison between the two techniques both on a larger scale than our study and in an in-home context [58]. Further study should be done to compare PE of Mosquito Shield™ or other indoor SR using CDC-LT and HLC in additional contexts and to explore potential biological or behavioral factors that may be driving the differences observed between techniques.

## Conclusion

HLC gave a similar estimate of PE of Mosquito Shield^™^ against *An. arabiensis* mosquitoes as the direct measurement of blood-feeding, while CDC-LT did not measure similarly to either blood-feeding or HLC and underestimated PE relative to the other techniques. The results of this study underscore that it is critical to first evaluate the use of CDC-LT (and other tools) in local settings prior to their use in entomological studies on the impact of indoor SR, and HLC remains the only practicable technique for measuring PE of SR in contexts in which direct measure of blood-feeding is not feasible.

## Data Availability

The datasets used and or analysed in this study are available from the corresponding authors upon reasonable request.
